# An Adaptive Wake-Up-Interval to Enhance Receiver-Based Ps-Mac Protocol for Wireless Sensor Networks

**DOI:** 10.3390/s19173732

**Published:** 2019-08-29

**Authors:** Mohammed Sani Adam, Lip Yee Por, Mohammad Rashid Hussain, Nawsher Khan, Tan Fong Ang, Mohammad Hossein Anisi, Zhirui Huang, Ihsan Ali

**Affiliations:** 1 Faculty of Computer Science & Information Technology, University of Malaya, Kuala Lumpur 50603, Malaysia; 2 College of Computer Science, King Khalid University Abha, Abha 61421, Saudi Arabia; 3 School of Computer Science and Electronic Engineering, University of Essex, Colchester CO4 3SQ, UK

**Keywords:** wireless sensor networks, wake-up radio, medium access control protocol, receiver-initiated MAC protocol, traffic adaptation

## Abstract

Many receiver-based Preamble Sampling Medium Access Control (PS-MAC) protocols have been proposed to provide better performance for variable traffic in a wireless sensor network (WSN). However, most of these protocols cannot prevent the occurrence of incorrect traffic convergence that causes the receiver node to wake-up more frequently than the transmitter node. In this research, a new protocol is proposed to prevent the problem mentioned above. The proposed mechanism has four components, and they are Initial control frame message, traffic estimation function, control frame message, and adaptive function. The initial control frame message is used to initiate the message transmission by the receiver node. The traffic estimation function is proposed to reduce the wake-up frequency of the receiver node by using the proposed traffic status register (TSR), idle listening times (ILTn, ILTk), and “number of wake-up without receiving beacon message” (NWwbm). The control frame message aims to supply the essential information to the receiver node to get the next wake-up-interval (WUI) time for the transmitter node using the proposed adaptive function. The proposed adaptive function is used by the receiver node to calculate the next WUI time of each of the transmitter nodes. Several simulations are conducted based on the benchmark protocols. The outcome of the simulation indicates that the proposed mechanism can prevent the incorrect traffic convergence problem that causes frequent wake-up of the receiver node compared to the transmitter node. Moreover, the simulation results also indicate that the proposed mechanism could reduce energy consumption, produce minor latency, improve the throughput, and produce higher packet delivery ratio compared to other related works.

## 1. Introduction

Wireless sensor networks (WSN) have gained a prolific attention in both academy and industry because of their wide-ranging applications, for example, health and remote monitoring/sensing [1,2]. WSNs are mostly deployed randomly in hostile and inaccessible areas. The main components of sensor nodes are a sensing unit, a control unit, a memory unit, and a limited battery power supply. The sensing unit is responsible for detecting the environment regarding humidity, temperature, and vibration. The control unit processes the sensed data and stores it into the memory unit of the sensor nodes. The sensor nodes are mainly powered by a battery with limited energy supply [3,4,5,6]. Research studies reveal that, in traditional WSN networks, the most energy consumption is done by the radio receiver. Furthermore, the radio receiver is monitored by the Medium Access Control (MAC) [7,8,9,10]. To utilize the wireless medium, MAC protocol was used to attain higher energy efficiency and low packet delivery latency. However, the leading bases of energy consumption in this protocol are idle listening and overhearing [11,12,13]. To reduced energy consumption, duty cycling has been widely used to design an energy-efficient MAC protocol [7,14]. Historically, the duty cycle mechanism has been proposed in MAC protocol, which falls into synchronous or asynchronous [11,15,16,17,18].

The synchronous mechanism coordinates neighboring sensor nodes to minimize energy consumption. Nonetheless, multi-hop time synchronization leads to huge overhead [19,20]. In disparity, the asynchronous duty cycle mechanism does not depend on any previous synchronization. Under a less congested traffic load, a huge number of the available asynchronous MAC protocols minimize energy consumption. However, the energy depletion in the synchronous duty cycling declines momentously with one or multiple communications and parallel traffic flows, and as a result, the synchronous does not scale well in huge and condensed networks [21,22].

Furthermore, in the duty cycling approach, sensor nodes periodically wake-up and check for an incoming message from the available channel. If there is no message received or sent, the sensor device changes from “active” to “sleep” mode to minimize energy consumption. Therefore, energy efficiency is the major concern in WSNs, and the MAC protocol handles most of the energy-related issues. The concern is to use the MAC protocol to improve the energy utilization of the WSN. As a result, designing a new protocol that reduces energy consumption is essential.

An Adaptive Wake-up-interval to enhance Receiver-based Preamble Sampling MAC protocol (AWR-PS-MAC) is proposed in this paper. Our proposed mechanism is designed for preventing the incorrect Traffic Status Register (TSR) convergence problem that causes the receiver node to wake-up more frequently than the transmitter node. The remainder of the paper is structured as follows. Relevant work of the receiver-initiated and the adaptive MAC protocols is described in Section 2. Then, background on the AWR-PS-MAC and the receiver-initiated MAC protocol discourse is given in Section 3. The methodology used and the simulation are explained in Section 4. The simulation results and evaluation through network simulations of the proposed mechanism compared with other related works are illustrated in Section 5. The final section of this paper presents the conclusion.

## 2. Related Works

Over the years, a large number of receiver-based PS-MAC protocols have been proposed by [23,24,25,26,27,28,29,30] to overcome the problem of a receiver node, which might wake-up twice or more compared to the transmitter node due to wrong traffic estimation. The following are the selected related works that have been analyzed based on strengths and weaknesses.

In [23], the authors proposed a protocol named Receiver-Initiated MAC (RI-MAC). In this protocol, when the receiver node is in an “active” mode, it broadcasts a preamble beacon message to the transmitter nodes. Then, the transmitter node starts sending the data message. Once the receiver node receives the data message, the receiver node sends another preamble beacon message. The preamble beacon message has two roles: (i) to acknowledge the data message was successfully received, and (ii) to notify the transmitter node that it is prepared to receive another data message. According to authors in [23], RI-MAC could minimize the energy depletion of the transmitter nodes because the transmitter nodes might only take part in transmitting a packet once they received the data message. However, the receiver node in this protocol uses a fixed wake-up interval and wakes-up twice or more compared to the transmitter node due to the incorrect TSR convergence because of the traffic pattern. Traffic patterns that are varied and unpredictable (variable traffic) might not be suitable to use with the fixed wake-up method, because this method might produce high latency for the transmitter nodes. 

In [24], the authors proposed a protocol named Predictive Wake-Up MAC (PW-MAC). In PW-MAC protocol, if the transmitter node has a data message to transmit, the transmitter node switches into an “active” mode and waits for a preamble beacon message from the receiver node. After receiving the preamble beacon message, the transmitter node starts sending the data message. Once the receiver node receives the data message, the receiver node sends another preamble beacon message. The preamble beacon message has three roles: (i) to acknowledgement the data message was successfully received, (ii) to inform the transmitter node that it is prepared to receive another data message, and (iii) to provide the current wake-up time to the transmitter node so that it can predict the future wake-up interval of the receiver node. According to the authors, PW-MAC could limit the Idle Listening Time of the transmitter nodes, because this method could forecast the wake-up interval of the receiver node. However, the prediction mechanism used in this method considered only the current wake-up time of the receiver node. Using only the wake-up interval of the receiver node causes more frequent wake-up than the transmitter node, because the prediction does not consider traffic estimation. To predict a traffic pattern that is varied and unpredictable (variable traffic), other parameters such as traffic estimation might need to be taken into consideration.

In [31], the authors proposed a protocol named Traffic-Aware Dynamic MAC (TAD-MAC). In this protocol, when the receiver node is in an “active” mode, it periodically broadcasts a preamble beacon message to the transmitter nodes. Then, the transmitter nodes only start sending the data message. Once the receiver node has received the data message, it aligns its wake-up interval based on the traffic rate of the transmitter node. Such information is kept at the specific register called the TSR. After that, the receiver node sends another preamble beacon message. This preamble beacon message has two roles: (i) to acknowledge the data message was successfully received, and (ii) to inform the transmitter node that it is prepared to receive another data message. According to authors, TAD-MAC could reduce overhearing and Idle Listening Time of the transmitter nodes, because the receiver node could use TSR to determine the wake-up interval for each of the transmitter node. However, the receiver node in this protocol might wake-up more frequently than the transmitter node. Moreover, the traffic pattern that is varied and unpredictable (variable traffic) might increase the overhead of the receiver node, because it might need to align its wake-up interval, often due to the fluctuation of the traffic data rate produced by the transmitter nodes. 

In [25], the authors proposed a protocol named Receiver-Initiated X-MAC with Tree Topology (TRIX-MAC). In this protocol, when the receiver node is in an “active” mode, it appends a new field into the preamble beacon message and periodically broadcasts it to the transmitter nodes. This field consists of the information about the number of time-slots that are required for each transmitter node to transmit their data message. The purpose of this field is to let the transmitter nodes forecast the next wake-up interval of the receiver node. According to the authors, TRIX-MAC could reduce energy consumption by enabling the transmitter nodes to forecast the receiver nodes’ wake-up times. Moreover, this protocol could decrease the number of data message exchanges between the receiver node and the transmitter nodes. However, the receiver node in this protocol might still wake-up more frequently than the transmitter node, because it is difficult and challenging to approximate the data transmission rate of each of the transmitter nodes.

In [32], a protocol named Heuristic Self-Adaptive MAC (HSA-MAC) was proposed. In this protocol, the receiver node uses open-loop and closed-loop to adapt its wake-up and sleep patterns. Open-loop is used to evaluate the behavior pattern of the static traffic rate, while closed-loop is used to bring up the next wake-up interval of the receiver node. According to the authors, HAS-MAC allows the receiver node to adapt its wake-up and sleep intervals in static and variable traffic rates. However, the feedback method used in the closed-loop needs more energy, especially in variable traffic networks, because the receiver node needs to wake-up more frequently than the transmitter node to predict and update its wake-up and sleep intervals.

In [26], a protocol named Adaptive Scheduling Predictive-Wakeup MAC (AS-PW-MAC) was proposed. AS-PW-MAC uses a similar predictive mechanism as in PW-MAC protocol, but there is an additional pseudo-random scheduling generator incorporated in the predictive wake-up mechanism. This pseudo-random scheduling generator is used to enable the transmitter node to forecast the wake-up time of the receiver node so that the transmitter node can wake-up before the receiver node. According to the authors, AS-PW-MAC is able to achieve a better packet delivery ratio in the maximum range of traffic loads compared to PW-MAC. However, AS-PW-MAC requires additional computation energy to forecast the wake-up time of the receiver node. Moreover, the pseudo-random scheduling generator might not be suitable to be used in variable traffic in WSNs, because the receiver node in AS-PW-MAC has to wake-up more frequently due to incorrect TSR convergence, and this protocol cannot detect traffic rate changes. 

In [27], a protocol named Prediction-Based Asynchronous MAC (PBA-MAC) was proposed. The receiver node in this protocol sporadically wakes-up to broadcast a preamble beacon message to inform the transmitter nodes to start the data message transmission. Upon receiving the preamble beacon message by the transmitter node, the transmitter node can send the data message to the receiver node. PBA-MAC used an advanced mechanism to enable the transmitter node to forecast the wake-up time of the receiver node. According to the authors, the use of an exponential advance mechanism in PBA-MAC could reduce the communication cost by allowing the transmitter node to forecast the wake-up time of the receiver node. Although the transmitter node can forecast the wake-up time of the receiver node using the exponential advance mechanism, the receiver node might not be able to coordinate its wake-up interval with the traffic rate of the transmitter nodes. Therefore, the receiver node might wake-up more frequently than the transmitter node, and this might lead to high energy consumption because of avoidable wake-ups of the receiver node.

In [30], a protocol named A Low Duty Cycle Efficient MAC Protocol Based on Self-Adaption and Predictive Strategy (AP-MAC) was proposed. In this protocol, an information table is used to store the receiver nodes and the neighboring transmitter nodes information. The transmitter nodes use the information table to control the wake-up time of the receiver node. The transmitter nodes wake-up to listen to a preamble beacon message from the receiver node. After receiving the preamble beacon message, the transmitter nodes set up a channel to transmit the data message. The authors claimed that this protocol could reduce the crosstalk problem. Crosstalk happens when there is communication interference between transmitter nodes. Nonetheless, AP-MAC protocol is not proper to be implemented in variable traffic, because the traffic changes are unpredictable. Therefore, this protocol might result in unnecessary wake-ups that incur extra energy consumption.

In [33], an adaptive contention window MAC protocol was proposed to provide better throughput under a heavy load. This protocol chooses from the history of the collision to reflect the communication status and the usage of the wireless channel. A huge collision reflects greater competition in the wireless channel, and a big contention window is needed based on this condition. The authors claimed to prolong the access time to get rid of the competition. However, this exponential increase in speed when the traffic load is dense could possibly lead to avoidable delays. Therefore, this protocol might lead to heavy traffic load that incurs latency.

In [34], a protocol named self-adaptive sleep/wake-up scheduling was proposed. In this scheduling protocol, a reinforcement learning technique is used to allow every sensor node to independently choose its own operation mode—sleep, listen, or transmission mode—and every time slot is in a distributed system. In this proposed protocol, the time axis is distributed into the time slots. However, in each of the time slots, each sensor node independently chooses to either sleep or wake-up. Furthermore, it is primarily focused on the abstract level with some assumptions that the problem addressed has not been solved. Therefore, the aforementioned problem still exists under these assumptions.

In [35], a novel receiver-initiated MAC protocol was proposed to improve the data delivery packet delay. In this protocol, all sensor nodes choose a different time slot for beacon message transmission in a distributed system in respect to their transmission range, and hop counts from the based station improve the data delivery delay. The proposed protocol extended RI-MAC to achieve the aforementioned property. In addition, the author extended the back-off mechanism to support control messaging. Differently from the RI-MAC, when a node attempts to send a control message, it sends the message instantly after the beacon reception. However, the receiver node in this protocol uses a time slot, and it may not be suitable for variable traffic patterns. A traffic pattern that is varied and unpredictable (variable traffic) might not be suitable to use with the time slot method, because this method might produce high delay.

In [36], the authors presented a novel MAC protocol for energy-harvesting based WSNs using the advantage of ultralow-power wake-up radios. To eliminate this issue of a small range of wake-up radios, more than one hop wake-up technique based on a two radio system is proposed for aiding the communications between destination and any sensor nodes while upholding low latency and minimal energy consumption. To minimize the energy depletion, wake-up calls and data packets are sent using two different data rates with the address of the destination node. Moreover, by spending high data rate for data transmission, it reduces the risk of data transmission collisions. However, only three sensor nodes are used for evaluating the performance. As a result, conclusions could not be made, because the investigation was not carried out in larger and denser networks. 

In [14], a Bird-MAC protocol for the Internet of Thing (IoT) applications was proposed that extremely minimizes the energy consumption of IoT applications in which sensor nodes report monitored status in a quasi-periodic manner, as in organized health-related and static environmental monitoring. The Bird-MAC protocol boasts extremely minimal energy consumption because it periodically sends the monitored information via a very low data rate. The high energy-saving impact of Bird-MAC (regardless of either a transmitter or a receiver) is because sensor nodes only wake-up for the duration of the actual clock drift among the transmitter–receiver pair, while the time duration of the wake-up is relative to the extreme clock drift. However, Bird-MAC requires additional computation energy to determine the maximum clock drift, and this protocol cannot be applied to variable traffic. 

## 3. Background on Receiver-Initiated MAC and the Proposed Mechanism

**Figure 1:**
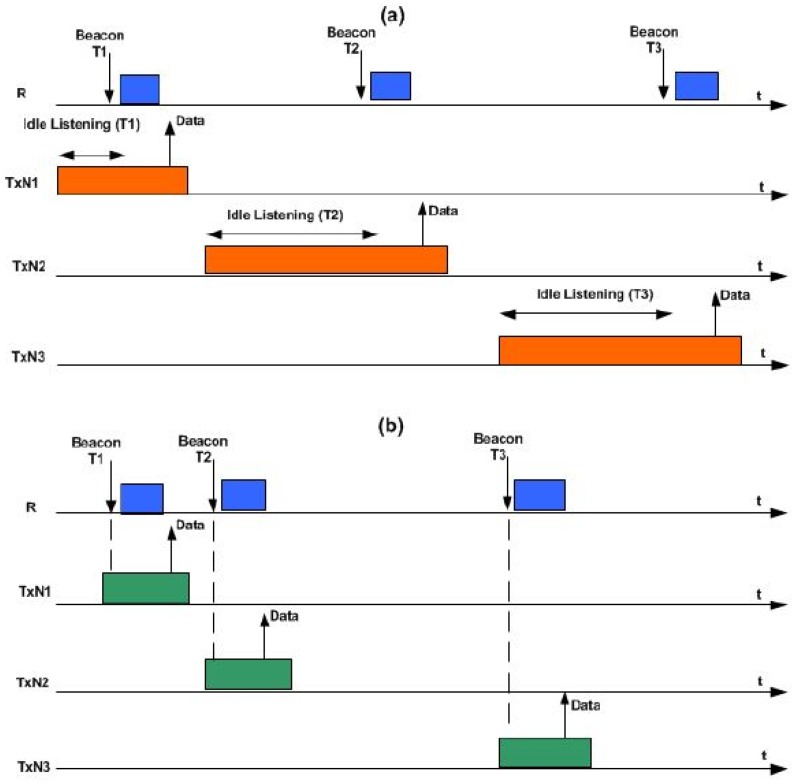
Receiver-initiated Preamble Sampling Medium Access Control (PS-MAC) general procedure [37]. (**a**) before convergence and (**b**) after convergence), and shows the communication of the three transmit nodes “TxN1, TxN2, and TxN3” trying to send data packet to a coordinating node (R). During the first phase (which we termed as an ‘evolution phase’), before reaching a steady state (Figure 1a), each TxNi will waits for the WUB message from the receiver node before sending its data packet. The wake-up beacon packet is implored to an explicit transmit node containing its unique node ID (identifier). Whereas, other intending transmit nodes continue to wait for their respective wake-up beacons time. After several wake-ups, the receiver node adapts its WUI time based on the traffic it receives from each of the transmit node. In the second phase (i.e., after reaching the convergence as shown in Figure 1b), the receiver node has adapted its WUI time in such a way that ILT is minimized. To accommodate for the clock drift and hardware latencies, the receive node sends the WUB message slightly after its scheduled time to guarantee that the anticipating transmit node is already awake.

The core idea of the receiver-based PS-MAC protocol is that the receiver node initiates the communication, assuming there is a single receiver node. In this protocol, the receiver node occasionally wakes-up and broadcasts a preamble beacon message to indicate that the receiver node is ready to accept a data message from any of the transmitter nodes. If no transmitter node has a data message to send, the receiver node switches back to the sleep “mode”. This protocol addresses the high delay issue that occurred at the other transmitter nodes that have data messages to send, because this protocol prevents the communication channel from occupying the preamble message sent by the transmitter nodes [25]. However, a receiver node that uses this protocol to transmit a packet might wake-up twice or more compared to the transmitter node due to wrong traffic estimation [32]. This situation could cause energy wastage and produce high latency. Therefore, this research work was carried out to overcome the aforementioned problem. Figure 1 elucidates the receiver-initiated PS-MAC protocols. Note that the main problem in receiver-initiated PS-MAC protocols is how the transmitter and the receiver node pair define there mutually agreed upon wake-up time. 

The figure is divided into two phases—before and after the convergence. In part (a), the receiver node periodically sends a Wake-Up Beacon (WUB) message to notify the transmitter nodes of its wake-up. In part (b), the transmitter node periodically wakes-up during its Wake-Up Interval (WUI) time, and the WUI is stored by the transmitter node. Before sending the control message, the transmitter node waits for the WUB from the receiver. This period is called Idle Listening Time (ILT), which is the activity that consumes the most energy in the receiver-initiated MAC protocols. After receiving the WB, the transmitter node sends the control message after sensing the medium. The communication ends with an acknowledgement (ACK) message from the receiver node to the transmitter node after it has successfully received the control message [37].

In this paper, we propose the AWR-PS-MAC designed to prevent the incorrect TSR convergence problem, which causes the receiver node to wake-up more frequently than the transmitter node. 

**Figure 2:**
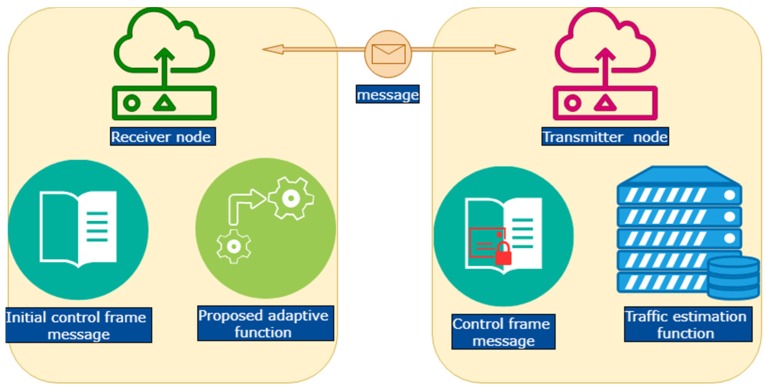
The main components of the proposed Adaptive Wake-Up Preamble Sampling MAC Protocol (AWR-PS-MAC).

Figure 2 exemplifies the main components of the proposed mechanism. The proposed mechanism adopts and amends the WUI time and the TSR proposed by [31]. The improvised WUI time and TSR are used to minimize the energy wastage and the high latency problems caused by the wake-up time of a receiver node due to wrong traffic estimation. To simplify the explanation, a receiver node and a transmitter node are used to illustrate how our proposed mechanism works. Our proposed mechanism has four components: Initial Control Frame Message, Traffic Estimation Function, Control Frame Message, and Adaptive Function.

### 3.1. Initial Control Frame Message

Initially, a receiver node initiates communication by sending an initial control frame message to a transmitter node. The initial control frame message consists of a WUB message with the size of two bytes. The WUB is a series of messages periodically sent by the receiver node to notify the transmitter node that there is a future data frame message. After every transmission, the receiver node waits for the transmitter node to respond.

#### Proposed Adaptive Function

Under the proposed adaptive function, the receiver node receives the Control Frame Message. The receiver node then replays an ACK message to the transmitter node. Then, the receiver node checks whether the WUB message is in an “active” mode. If the WUB message is in the “active” mode, the receiver node uses the proposed Adaptive Function stated in Equation (1) to calculate the WUI time. Otherwise, Equation (2) is used.

(1) $$WUI_{t}\left(n+1\right)=\overset{n}{\underset{i=j}{\sum }}\frac{WUI_{t}\left(j\right)+ILT_{n}-ILT_{k}}{NW_{wbm}+1}$$

(2) $$WUI_{t}\left(n+1\right)=\overset{n}{\underset{i=j}{\sum }}\frac{WUI_{t}\left(j\right)+N_{0}\left(i\right)\;*\;t_{ref}}{NW_{wbm}+1}$$

*ILT_n_* and *ILT_k_*, represent the Idle Listening Time, and *n* and *k* are auto-increment variables, which are used to keep track of the first two successful data packet transmissions. *ILT_n_* and *ILT_k_* are the ILTs for the first two successful data packet transmissions. *NW_wbm_* represents the “Number of Wake-up without Receiving Beacon Message” and is used to store the statistical information of the received WUB Message. *N*_0_ is the number of occurrence of zeros in the TSR for a transmitter node, tref is the simulation time, and *j* and *i* are two successful data packets received.

In the current system, the Adaptive Function cannot detect the changes in the traffic rate if the traffic rate is high [24,31]. In the proposed AWR-PS-MAC protocol, the receiver node adapts its WUI time based on the traffic information received using the proposed Adaptive Function. The proposed Adaptive Function uses the traffic information collected to reduce the wake-up frequency of a receiver node to minimize the energy depletion as well as the message overhead.

After going through the proposed Adaptive Function, the receiver node learns about the traffic information of each transmitter node. The receiver node updates the TSR corresponding to all the transmitter nodes. After that, the receiver node stores the transmitter node identification number, the WUI time, and the ILT. For the next data packet transmission, the receiver node uses the latest TSR information to determine the next WUI time for a transmitter node. With the proposed mechanism, the receiver node wakes-up close to the transmitter’s WUI time. Thus, the proposed mechanism can reduce energy consumption as well as the message overhead.

### 3.2. Transmitter Node

The transmitter node consists of two functions—control frame message and traffic estimation function (see Figure 2). Below are the explanations of each of the functions.

#### 3.2.1. Control Frame Message

After going through the proposed traffic estimation function, the transmitter node then transmits the Control Frame Message to the receiver node. The Control Frame Message consists of Frame Control, Address Information, Idle Listening Time, “Number of Wake-up without Beacon Message,” Data Payload Field, and Check Sum fields (see Table 1). The Frame Control field belongs to the MAC header section. It has 1 byte. It also contains the type of message used by the proposed mechanism, such as WUB, Data Packet, and ACK. The Address Information field has 4 bytes. It stores the address information of the destination and source nodes. ILT has 1 byte. It is used to store the listening time when a sensor node is in “active” mode. The “Number of Wake-up without Beacon Message” field has 1 byte. It stores the information of the number of times the transmitter node wakes-up before it receives the WUB message. The Data Payload field has a variable data size. It holds the actual data to be sent by the transmitter node. The Check Sum has 2 bytes. It is used to determine whether an error has occurred during the data packet transmission. The receiver node then calculates the next wake-up interval time using the Adaptive Function.

#### 3.2.2. Traffic Estimation Function

In the current approach, the process of storing traffic information does not consider the data rate of the transmitter node. An incorrect TSR convergence problem might occur when the receiver node wrongly predicts the WUI time based on the TSR record that stores the traffic information of the sensor nodes. Therefore, it makes the receiver node wake-up more frequently than the transmitter node. To overcome this problem, a traffic estimation function is proposed.

After the transmitter node has received the initial control frame message, the transmitter node calculates ILTn, ILTk, and NWwbm. n and k are auto-increment variables, which are used to keep track of the first two successful data packet transmissions. ILTn and ILTk are the ILTs for the first two successful data packet transmissions. NWwbm is the “Number of Wake-up without Receiving Beacon Message” from the receiver node. NWwbm is used to store the statistical information of the received WUB message. This information is used to prevent the incorrect TSR convergence problem that can cause the receiver node to wake-up more frequently than the transmitter node. 

## 4. Simulation Experiments

Sensor nodes are randomly deployed to evaluate the proposed techniques using the parameters shown in Table 2. We used Objective Modular Network Testbed in C++ (OMNeT++) and mixed simulator (MiXiM) to simulate the transmission of the packets. Table 2 shows the simulation parameters and the values used for simulating the proposed mechanism under variable traffic. To have a fair comparison with the benchmark work, the configuration settings that are commonly used for simulating variable traffic were adopted from [32]. The simulation was run for a period of 2000 s, where 5 to 50 nodes were randomly deployed in an area of 500 m^2^. The WUI time was set from 0.5 s to 2 s, and each of the simulation results were calculated after 100 stochastic simulations. The traffic rate used was based on the increase or the decrease of the ILT of the sender nodes, and the traffic rate range was from 1 to 10 frames/s. The TSR length used was set to 1 byte for storing the values of messages received by the sender node. One receiver node was used, and the constant bit rate (CBR) traffic model was used to generate traffic. 

## 5. Result and Discussion

### 5.1. The Performance Analysis for Energy Consumption Proposed a Method and Other Related Works

The energy consumption is calculated using Equation (3). This equation is adopted from [38]. It is the standard formula used for calculating the energy consumed.

(3) $$E=E_{tx}^{WUB}+E_{rx}^{WUB}+E_{tx}^{d}+E_{rx}^{d}+E_{sam}+E_{oh}+E_{s}+E_{i}$$

where $E_{tx}^{WUB}$ is the amount of energy used in transmitting WUB. $E_{rx}^{WUB}$ is the amount of energy used in receiving WUB. $E_{tx}^{d}$ is the amount of energy that is essential to communicate a data packet. $E_{rx}^{d}$ is the amount of the energy required to receive a data packet. $E_{sam}$ is the amount of energy necessary to sample the channel for an ongoing transmission. $E_{oh}$ is the energy of overhearing when a sensor node overhears a message that is destined to another sensor node. $E_{s}$ is the amount of energy used when the mode of the sensor is in “sleep mode”. $E_{i}$ is the energy of ILT.

**Figure 3:**
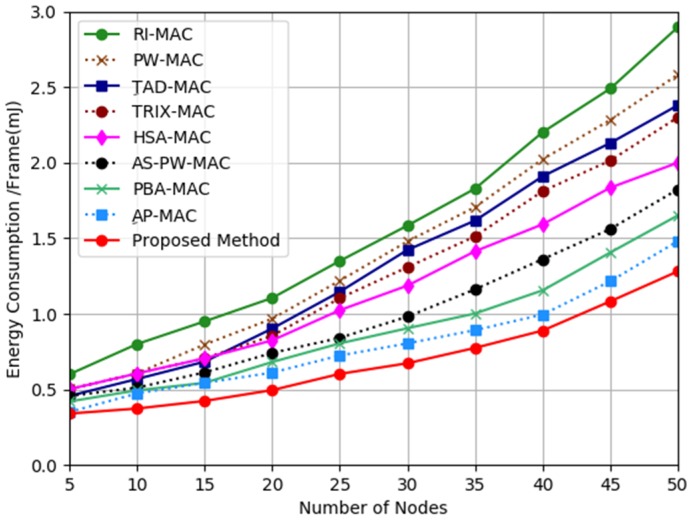
Energy consumption analysis.

Figure 3 illustrates the energy consumption analysis results from the comparison among the proposed method, AP-MAC, PBA-MAC, AS-PW-MAC, HSA-MAC, TRIX-MAC, TAD-MAC, PW-MAC, and RI-MAC. The figure illustrates that the proposed mechanism had a very significant decrease in energy consumption compared to other related works. On average, the proposed method produced approximately 14%, 25%, 34%, 43%, 56%, 60%, 67%, and 77% less energy consumption compared to AP-MAC, PBA-MAC, AS-PW-MAC, HSA-MAC, TRIX-MAC, TAD-MAC, PW-MAC, and RI-MAC, respectively.

On average, AP-MAC protocol produced the second least energy consumption compared to other related works. AP-MAC protocol managed to consume less energy because it utilized the information table to know the wake-up time of the receiver node. However, this protocol was still less effective than the proposed mechanism except for the number nodes at five (which tied with the proposed method).

On average, PBA-MAC protocol produced the third least energy consumption compared to other related works. However, it provided the same energy consumption as AP-MAC for the number nodes at 10 and 15. The reason that PAB-MAC managed to deliver less energy depletion compared to other protocols was that it used an advance mechanism to enable the transmitter node to forecast the wake-up time of the receiver node. However, this protocol still produced higher energy consumption compared to the proposed mechanism.

Generally, AS-PW-MAC protocol produced the fourth least energy consumption compared to other related works. However, it produced the same energy consumption as PBA-MAC protocol for the number nodes at 5 and 10. The reason that AS-PW-MAC managed to deliver less energy depletion compared to other protocols was that it used a pseudo-random scheduling generator to enable the transmitter node to forecast the wake-up time of the receiver node so that the transmitter node could wake-up before the receiver node. However, this protocol still consumed high energy depletion compared to the proposed mechanism. 

TAD-MAC and TRIX-MAC protocol produced the fifth least energy consumption compared to other related works for the number nodes at 5 until 15. TRIX-MAC protocol dropped to sixth place when the number of sensor nodes increased to 20 until 50. The reason that TRIX-MAC managed to produce less energy depletion compared to other protocols was that TRIX-MAC enabled the transmitter nodes to forecast the receiver nodes’ wake-up times. On the other hand, TAD-MAC protocol dropped to seventh place when the number of sensor nodes increased to 20 until 50. The reason that TAD-MAC protocol could not perform well compared to other protocols was that the receiver node in this protocol woke-up more frequently than the transmitter node. 

HSA-MAC protocol and PW-MAC protocol produced the sixth least energy consumption compared to other related works for the number nodes at 5 until 10. PW-MAC protocol started to drop to seventh place for the number nodes at 15 and then to second to last place for the number nodes at 20 until 50. The reason that PW-MAC protocol produced higher energy depletion when the number of nodes increased was due to the prediction mechanism used in this protocol that made the receiver node wake-up more frequently than the transmitter node. Ironically, HSA-MAC protocol not only managed to retain sixth place for the number nodes at 15, but it also managed to improve its place, moving to fifth place for the number nodes at 20 until 50. The reason that HAS-MAC could reduce the energy depletion when the number of nodes increased was that this protocol allowed the receiver node to adapt its wake-up and sleep intervals in static and variable traffic rates.

In general, RI-MAC protocol produced the highest energy consumption compared to all the related works. The reason that RI-MAC protocol produced the highest energy consumption was that this protocol used a fixed wake-up interval, which made the receiver node wake-up more frequently than the transmitter node. 

To recap, the results from the simulation show that the proposed mechanism can outperform other related works. This means that the proposed mechanism that uses ILT and NWwbm can prevent the incorrect TSR convergence problem that causes the receiver node to wake-up more frequently than the transmitter node.

### 5.2. The Performance Analysis for Energy Consumption Proposed a Method and Other Related Works

Equation (4) is used to determine the latency of all the protocols. This equation is the standard formula, and it is adapted from [38].

(4) $$L=\frac{\sum_{i=1}^{P_{rx}}\left(T_{rxi}-T_{pgi}\right)}{P_{rx}}$$

where L is the latency. $P_{rx}$ is the packet received. $T_{rxi}$ is the time when the packet is received. $T_{pgi}$ is the time when a generated packet is received.

**Figure 4:**
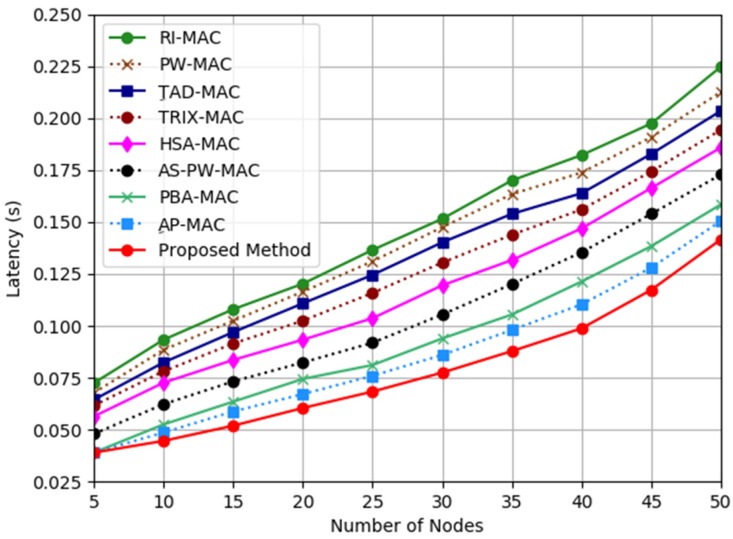
Latency analysis.

Figure 4 shows the latency analysis results from the comparison among the proposed mechanism, AP-MAC, PBA-MAC, AS-PW-MAC, HSA-MAC, TRIX-MAC, TAD-MAC, PW-MAC, and RI-MAC. In general, the latency increased when the number of nodes increased for all the protocols. However, the proposed mechanism had the least latency compared to AP-MAC, PBA-MAC, AS-PW-MAC, HSA-MAC, TRIX-MAC, TAD-MAC, PW-MAC, and RI-MAC. The proposed mechanism reported 6%, 11%, 19%, 26%, 31%, 35%, 40%, and 45% lesser latency compared to AP-MAC, PBA-MAC, AS-PW-MAC, HSA-MAC, TRIX-MAC, TAD-MAC, PW-MAC, and RI-MAC, respectively. This analysis shows that the proposed mechanism can reduce the latency for the variable traffic by using the proposed adaptive function. The proposed adaptive function uses the latest TSR information to determine the next WUI time for the transmitter’s WUI time. Therefore, it can help the receiver node to adapt its WUI time based on the traffic information received.

### 5.3. The Performance Analysis for Energy Consumption Proposed a Method and Other Related Works

A standard formula for calculating the throughput is adopted from [39] to determine the throughput of all the protocols. Hence, Equation (5) is used.

(5) $$T=\frac{N_{rp}S_{p\;}}{T_{s}}$$

where T is the throughput. $N_{rp}$ is the number of packets received. $S_{p}$ is the size of the packet. $T_{s}$ is the simulation time.

**Figure 5:**
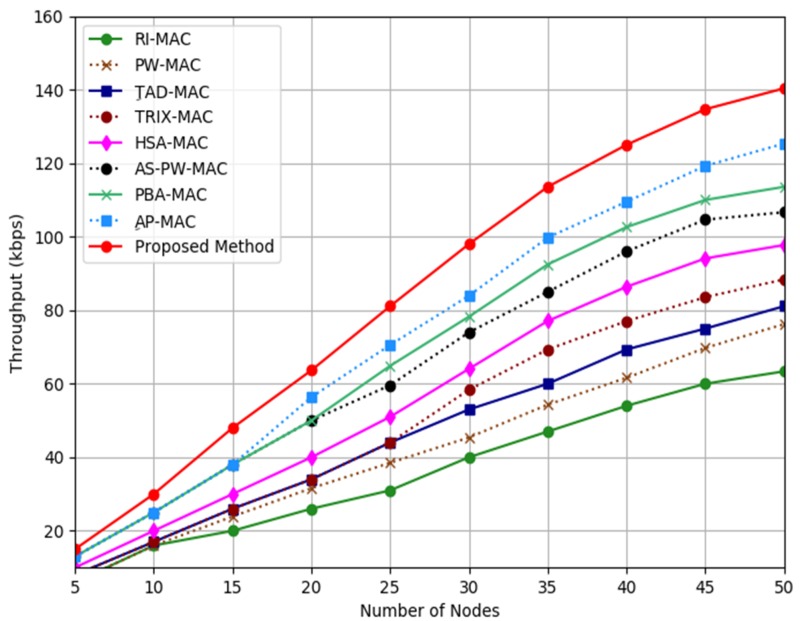
Throughput analysis.

Figure 5 shows the throughput results from the analysis for the proposed mechanism, AP-MAC, PBA-MAC, AS-PW-MAC, HSA-MAC, TRIX-MAC, TAD-MAC, PW-MAC, and RI-MAC. The throughputs of the proposed mechanism, AP-MAC, PBA-MAC, AS-PW-MAC, HSA-MAC, TRIX-MAC, TAD-MAC, PW-MAC, and RI-MAC showed a linear increment when the number of notes increased. From the graph, the throughput produced by the proposed mechanism was about 11%, 21%, 27%, 36%, 46%, 54%, 59%, and 74% higher than AP-MAC, PBA-MAC, AS-PW-MAC, HSA-MAC, TRIX-MAC, TAD-MAC, PW-MAC, and RI-MAC respectively. The reason that the proposed mechanism can outperform the other methods is that the proposed adaptive function uses WUB message to notify the transmitter node so that the receiver node is ready to receive any incoming data packet. With this modification, it enables the proposed mechanism to produce higher throughput.

### 5.4. The Performance Analysis for Energy Consumption Proposed a Method and Other Related Works

The packet delivery ratio is calculated using Equation (6). This equation is the standard formula used by [39] to compute the packet delivery ratio.

(6) $$PDR=\frac{P_{rx}*100}{\sum_{i=1}^{n}P_{g}}$$

where PDR is the packet delivery ratio. $P_{rx}$ is the number of packets received. n is the number of sensor nodes. $P_{g}$ is the total number of the packet generated.

**Figure 6:**
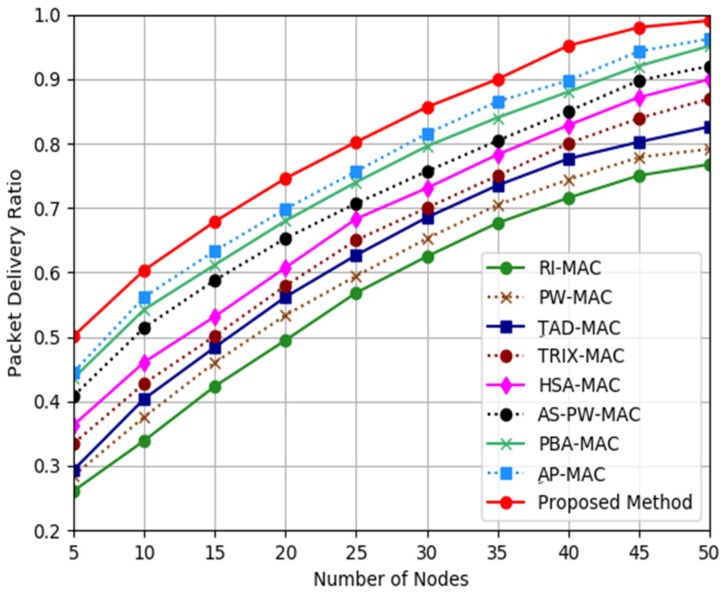
Packet Delivery Ratio analysis.

Figure 6 presents the results with respect to the Packet Delivery Ratio for the proposed mechanism, AP-MAC, PBA-MAC, AS-PW-MAC, HSA-MAC, TRIX-MAC, TAD-MAC, PW-MAC, and RI-MAC. In general, the results obtained from the simulation demonstrated that the proposed mechanism had a higher Packet Delivery Ratio compared to AP-MAC, PBA-MAC, AS-PW-MAC, HSA-MAC, TRIX-MAC, TAD-MAC, PW-MAC, and RI-MAC. The Packet Delivery Ratio of the proposed mechanism was about 7%, 10%, 13%, 18%, 22%, 25%, 44%, and 54% higher compared to AP-MAC, PBA-MAC, AS-PW-MAC, HSA-MAC, TRIX-MAC, TAD-MAC, PW-MAC, and RI-MAC, respectively. The reason that the proposed mechanism can provide better Packet Delivery Ratio is that the proposed adaptive function uses ILT_n_, ILT_k,_ and NW_wbm_ to schedule the receiver node so that the transmitter node can receive an incoming data packet in a shorter waiting time.

The simulation results show that, on average, the proposed mechanism can outperform other related works in terms of energy consumption, latency, throughput, and Packet Delivery Ratio by 14%, 6%, 11%, and 7%, respectively. The reason that the proposed mechanism can achieve better results is because the proposed Adaptive Function uses the latest TSR information to determine the next WUI time for the transmitter’s WUI time. Therefore, it can help the receiver node to adapt its WUI time based on the traffic information received and prevent the incorrect TSR convergence problem that causes the receiver node to wake-up more frequently than the transmitter node.

## 6. Conclusions

The AW-RB-PS-MAC protocol was proposed. The proposed AW-RB-PS-MAC protocol consists of four components: Initial Control Frame Message, Traffic Estimation Function, Control Frame Message, and Adaptive Function. Initial Control Frame Message is the initial message sent by a receiver node. It consists of WUB message and has two bytes. Traffic Estimation Function is used by a transmitter node to estimate the traffic rate by using the TSR and the proposed variables. Control Frame Message consists of Frame Control, Address Information, Idle Listening Time, “Number of Wake-up without Beacon Message,” Data Payload Field, and Check Sum fields. The Frame Control field belongs to the MAC header section. It has 1 byte. The Address Information field has 4 bytes. Idle Listening Time (ILT) has 1 byte. The “Number of Wake-up without Beacon Message” field has 1 byte. The Data Payload field has a variable data size. The Check Sum has 2 bytes. Finally, the receiver node to calculate the next WUI time of each of the transmitter nodes uses the Adaptive Function. The proposed Adaptive Function uses the proposed function in two ways. When the WUB is in “active” mode, Equation (1) is used; otherwise, Equation (2) is used. Furthermore, the proposed Adaptive Function uses new features to reduce energy consumption and high latency. The implementation of the proposed AW-RB-PS-MAC protocol was carried out in the OMNeT++ network simulator and the MiXiM framework. The results showed that the proposed mechanism could prevent the incorrect TSR convergence problem that causes the receiver node to wake-up more frequently than the transmitter node.

In the future, we will conduct research on preventing other issues, such as wake-up collision [32] and data packet collision [26] in variable traffic. The wake-up collision occurs when one or more transmitter nodes share the same wake-up time while they are within transmission range of each other. This means that these transmitter nodes send a wake-up beacon at the same time to the receiver node and collide in the transmission channel. This problem causes more energy consumption at the transmitter nodes. Data packet collision happens when more than one transmitter node sends a data packet concurrently to the receiver node using the same channel. This problem causes more packet retransmission and high energy consumption at the transmitter nodes. We believe that, by looking into these issues, the adaptation of the variable traffic method could be improved.

## Figures and Tables

**Table 1:** Control Frame Message (send by transmitter node).

Description	Size (Bytes)
Frame Control	1
Address Information	4
Idle Listening Time	1
Number of Wake-up without Beacon Message	1
Data Payload	Variable
Check Sum	2

**Table 2:** The simulation settings.

Field	Values
Simulation Time	2000 s
Number of Nodes	Data
Node Distribution	Randomly
WUI time	0.5 s–2 s
Number of Ransom Simulation	100
Traffic Rates	1–10 frames/s
Bitrate	250 kbit/s
Rx (receive) Current	18.8 mA
Tx (transmit) Current	17.4 mA
Sleep Current	0.03A
Number of Receiver	1
Traffic Model	Constant Bit Rate
